# 

**Published:** 1872-01

**Authors:** 


### THE GEORGIA

### MEDICAL COMPANION.

EDITORS:

T. S. POWELL, M. D., and W. T. GOLDSMITH, M. D.

ASSOCIATE EDITORS:

GEORGIA.

Robert Battey, M D,

R C Word, M D,

W L Kirkpatrick, M D,

James A Long, M D,

W L M Harris, M D,

H L Battle, M D,

W C Musgrove, M D,

L B Bouchelle, M D,

John C Drake, M D,

E F Knott, M D,

Thomas Burdell, M D,

E H W Hunter, M D,

V S Hitt, M D,

J E Pope, M D,

C H Bass, M D,

J A Hunnicutt, M D,

A H Brantley, M D,

J J Knott, M D,

J C Wisdom, M D,

W W Leake, M D,

ALABAMA.

J C Knox, M D,

Geo F Taylor, M D,

J B Barnett, M D,

S M Hogan, M D.

D. S Hopping, M.D.

J T Searcy, M.D.

J F Hill, M.D.

MISSISSIPPI.

C B Gallaway, M D,

Edward Lea, M D,

S V D Hill, M D,

W B Harvey, M D,

J W M Shattuck, M D,

E T Henry, M D,

T P Lockwood, M D,

Robert Kells, M D.

R J Preston, M D,

A D Hodge, M D, Ark.

VIRGINIA.

Charles S Lewis, M D,

John H Claiborn, MD,

F Horner, Jr, M D,

A S Payne, M D,

J. E. Lockridge, M. D.

J S Whorton, M D.

Wm O Owen, M D,

Sam'l P Ripley, M D,

Benj Blackford, M D,

E A Drewry, M D,

Rob. B Tunstall, M D,

A J Terrell, M D,

W. F Barr, M D,

L. B. Anderson, M. D.

S L Jepson, M.D., W Va

J M Lazzell, M. D.,

J K Hodge, M D, Ark

SOUTH CAROLINA.

E T McSwain, M D.

G M Rivers, M.D.,

J. D. Tucker, M. D., Tenn.

Swan M Burnett, M. D., Tenn.

S M Welch, M D, Tex

T J Heard, M D, Tex.

W A Heard, M D, Tex.

NORTH CAROLINA.

Geo A Foote, M D.

Thos. F Wood, M D.

H Kelly, M. D.

J R Hughes, M. D.

N J Pittman, MD,

S S Satchwell, M D.

A B Pierce, M D,

E A Anderson, M D

H T Bahnson, M. D.

Willis Alston, MD,

CORRESPONDING EDITORS:

J M Toner, M D, Washington City, D C; John H Weir, M D, Ill; Thomas J Gallaher, M D, Pa; Ely Van DeWarker, M D, N Y; Rob't Robson, M.D. Ind; C C F Gay, M D, N Y, (Surgeon of Buffalo General Hospital); W T Taylor, M D, Ky; A Patton, M D, Ind; J Orne Green, Boston, Mass; B W Stone, M D, Ky, (Assistant Physician Western Lunatic Asylum); James Tyson, M D, Philadelphia, Pa; M H Henry, M D, N Y, (Surgeon to New York Dispensary, Department of Venerial and Skin Diseases); Wm R Whitehead, M D, N Y; Thomas H Kearney, M D, Cincinnati, O; Benjamin Howard, M D, New York, Chas Kearns, M D, Ky, S C Busey, M D, Washington, D C.; A W Hobson, M. D., Ark.; A W Calhoun, M. D., now of Berlin, Prussia; T Curtis Smith, O; George Strawbridge, M D, W W Leen, M D, Philadelphia.

"*QUICQUID PRÆCIPIES ESTO BREVIS.*"

ATLANTA, GA.
FRANKLIN STEAM PRINTING HOUSE.
J. J. TOON, PROPRIETOR.

TERMS, - - - $2 50 A YEAR, IN ADVANCE

Vol. 2. 1872

LIBRARY S. G. O.

Surgeon General's Office

53378

Washington, D.C.

W1
GE442G

### CONTRIBUTORS TO VOLUME II.

Robert Battey, M. D............Georgia.
L. B. Bouchelle, M. D............Georgia.
Swan M. Burnett, M. D............Tennessee.
A. W. Calhoun, M. D............Berlin, Prussia.
Thos. A. Cooke, M. D............Louisiana.
A. Donald, M. D............Louisiana.
—— Foster, M. D............Georgia.
C. C. F. Gay, M. D............New York.
C. B. Galloway, M. D............Mississippi.
E. T. Henry, M. D............Mississippi.
George D. Hodge, M. D............Arkansas.
J. Knox Hodge, M. D............Arkansas.
S. L. Jepson, M. D............West Virginia.
J. C. Kurt, M. D............Alabama.
E. W. Lane, M. D............Georgia.
J. M. Lewis, M. D............Mississippi.
W. L. Lipscomb, M. D............Mississippi.
Therodore P. Lockwood, M. D............Mississippi.
John E. Lockbridge, M. D............Virginia.
A. A. Lyon, M. D............Mississippi.
Thomas H. Mayo, M. D............Mississippi.
Wm. A. B. Norcom, M. D............North Carolina.
Alban S. Payne, M. D............Virginia.
R. J. Preston, M. D............Virginia.
Robert Robson, M. D............Indiana.
S. S. Satchwell, M. D............North Carolina.
J. W. M. Shattuck, M. D............Mississippi.
T. Curtis Smith, M. D............Ohio.
B. W. Stone, M. D............Kentucky.
W. T. Taylor, M. D............Kentucky.
George F. Taylor, M. D............Alabama.
J. D. Tucker, M. D............Tennessee.
E. M. Vasser, M. D............Alabama.
Ely Van de Warker, M. D............New York.
David Webster, M. D............New York.
John H. Weir, M. D............Illinois.
J. S. Wharton, M. D............Virginia.
R. C. Word, M. D............Georgia.

### INDEX:

—o—

Abscess of Vagina and Uterus, 102.
A case of paralysis of mortor portion of 7th nerve, 10.
A case of Glanders, 108.
A case of Calculus Nephritis, 39.
Acetate of lead in cavity of Lung, 680.
Acid of tomato, 700.
Acne pustulosa. 360.
Aconite to children, 33.
" productive of uterine irritation, 109.
" in lichen and prurigo, 688.
Addison's disease, 150.
A dressing for fracture, by B. W. Stone, M. D., 651.
Agency of mind in disease, 698.
A good local anæsthetic, by Dr. J. K. Hodge, 476.
Air of houses, 622.
Albuminuria, strychnia in, 167, 169.
" cathartic in, 488.
Alcohol, 364.
Alcoholic treatment of wounds, 149.
Alterative and anti-spasmodic mixture, 34.
Amenorrhœa and dysmenorrhœa, 22.
" treated by electricity, 96.
Amaurosis, 98.
Amyloid disease prevented, 69.
Anasarca formula for, 411.
" Surgical treatment of, 682.
A new adhesive substance, 697.
A new excepient for pills, 102.
Anodyne alterative, by Dr. George D. Hodge, 681.
Aneurism, 681.
Angina, treatment of scrofulous, 689.
Anhydrous glycerine, 113.
Anti-sceptic cere-cloth, 46.
Anti-neuralgia powder, 159.
Antidote to liquor poisoning, 239.
" for arsenic, 681.
Anus, ointment for itching of, 758.
Aperient and alterative formula, 40.
Aphoria, 36.
Apiol, 155.
Arsenical powder, 621.
Arsenic in irritable stomach, 359.
Artesian wells. by W. L. Lipscomb, M. D., 1.
Art for prescribing for children, 304.
A sovereign anti-bilious pill, by J. Knox Hodge, M. D., 94.
Asthma, nitrate of amyl in, 316.
" cured by rubbing, 475, 621, 771, 764.
Asthmatic bronchitis, chloral in, 759.
Aural polyp, 96.

Bad air, 743.
Bathing, rules for. 678.
" hints for sea, 766.
Bath nitro, muriatic, 753.
Bed-sores, treatment of, 305.
" " nitrate of silver in, 682.
Beef tea, 362.
Belladonna as an anti-gallactic, 307.
Benzoating ointment extemporaneously made, 45.
Benzoic acid in anal fistula, 46.
Bladder, ergot in paralysis of, 489.
Blenorrhagia, treatment of, 485.
Bloody discharges after micturition, 35.
Bone-felon arrested, 611.
Braun's colpeurynter as a tampon, 310.
Bread made with sea-water, 691.
Bromide of sodium, 26.
" Calcium, 160.
" Ammonim in dysmenorrhœa, 275
" of potassium, hysterical gasralgia consequent on a change of life, by Dr. Geo. F. Taylor, 400.
" Potassium, bad effects of, 417 496.
Bromine, an oxydizing agent, 744.
Bromo-chloralum, 744.
Bronchitis, seneca in, 768.
Broncho-pneumonia with typhoid symptoms, 98.
Brown water. formula, 692.
Bubo, treatment of, 153.
" caustic potosh for, 495.
Bullock's blood, a new remedy, 614.
Burns, a remedy for, 169, 684.

Calabar-bean in excitement of cerebro-spinal disease, 107.
Calculi, biliary, 164.
Calculus nephritis, 613.
Cancer, treated by electrolysis, 152.
Cancer, tannic acid in, 494.
" of tonsils, 761.

Cancrum oris, 94, 560, 768.
Canula, for tracheotomy, 362.
Carded wool in wounds, 152.
Carbolic acid preparations, 42.
" " in pitting of small-pox, 48
" " inhalation in phthisis, 303
" " causing gangrene, 430.
" " range of, 56.
" " antidote for, 684.
Carbuncle treated by cups, 340.
Carminative powder for infants, 161.
" mixture, 175.
Case of foreign body external to knee-joint, by T. Curtis Smith, M. D., 270
Castor oil emulsion, 749.
Catarrhus vessicus, formula for, 172.
Cateract. operation for, 163.
Catheter, danger of gutta-percha, 692.
" Sayre's vertebrated, 365.
Causes and treatment of obesity, by W. T. Taylor, M. D., 129.
Causes of liver complaint, 769.
Celery, a nervine, 361.
Cerebro-spinal meningitis, 97. 228, 426, 491, 607, 690, 759.
Cervical abscesses in children, 390.
Chancre, chloral for, 687.
Chilblains. 13, 47, 413.
Charcoal in ulcer of stomach, 109.
" in gastric & uterine diseases, 364.
Chloral in infantile convulsions, by L. B. Bouchelle, M. D., 19.
Chloral to infants, 34.
" in toothache, 157, 159, 479, 611.
" in genito-urinary diseases, 406.
" in the opium habit, 314.
" in venereal ulcer, 486.
" in chest diseases, 494.
Chlorate of potas., in dysentery, 105, 716
Chloride of Soda, 757.
Chloroform and glycerine, 100.
" antidote for poisoning, 616.
" in labor, 404.
" syrup of, 683.
Chlorosis, 113.
Chlorosis, Woode's formula, 419.
" formula for, 491.
Cholera infantum, 47, 48, 255, 701.
" chloral in, 310,
" a remedy for, 683, 759.
" quinine in, 685.
" latest cure for, 547.
Chorea, 47, 406, 463, 769.
" sulphate of zinc in, 158.
" calabar bean in, 611.
Chronic bronchitis treated by atomized turpentine-water, 350.
" " formula for, 114, 494.
" catarrh, formula for, 50.
Chronic cystitis, 100.
" chills, by Dr. J. D. Tucker, 472.
" diabetes, treatment of, 306.
" diarrhœa, sequel of typhoid fever, 35.
" cystitis, peroneal cystotomy, by Robert Battey, M. D., 328, 598.
" enlargement prostate glands, 106.
" otorrhœa and its cure by alcohol, by A. W. Calhoun, M. D., 88.
" do by David Webster, M. D., 136.
" otitis, 96.
Cocoanut milk, 617.
Cod-liver oil and arsenic formula, 36.
" " pepsine & pancreatine, 161
" " taste disguised, 312, 615, 682.
" " perfumed, 560.
Cold water as an oxcytoccic, 36.
" food for infants, 480.
Colds, cure for, 483.
Colic in babies, 422.
" 481.
Collodion, paint, 48.
" paper, 158.
Colliquative sweats, 420.
Compound syrup of assafœdta, 45.
Complete anchylosis of hip-joint, by Robert Battey, M. D., 271.
Congenital contraction of vagina, 44.
Conjunctivitis chronic, 605.
Concentrated emulsion of almonds, 691.
Connection of variolous diseases, 314.
Constipation, pill for, 478, 496.
" kneading in, 21.
Copabia, a new mode of giving, 240.
Correspondence, Dr. J. H. Weir, 275.
" Dr. T. P. Lockwood, 272.
" Dr. E. T. Henry, 340.
" Dr. E. W. Lane, 465.
" Dr. E. M. Vasser, 654.
Corns, a remedy, 608.
" cure for, 100.
Coryza, 613.
Cordee, Van de Court's pill for, 173.
Cotton-root in menstrual suppression 109
" " an anodyne, 110.
" " glycerized in wounds, 484.
Cough syrup, Jackson's, 422.
Counter irritant, 769.
" " new, 22.
Croton, chloral hydrate, 150, 476.
Croup, 171, 488, 560, 602.
Cyanoses, a remedy for, 485.
Cystolgia, 175.
Cystitis, formula for, 176.

Death, a positive sign of, 740.
" from syrup of poppies. 312.

Deafness, creasote in, 745.
Deficient secretion, 159.
Depression, strichnia & phosphoric acid in, 622.
Delfracee's revulsive liniment, 683.
Delirium tremens, 239.
Diabetes, tannic acid in, 622.
" ammono, saline treatment, 237.
" diet in, 237, 624, 676. 693.
" insipidus, 613.
Diagnosis of Bright's disease, 112.
" of fracture of neck of femur, 764.
Diaphoretic & expectorant mixture, 409.
Diarrhœa, pepsine in, 610.
Digitalis, 235, 236.
Diphtheria, 34, 157, 167, 427.
Diphtheretic enteritis, 311.
Diseases of rectum simulating uterine affections, 286.
Disinfectant, 751.
Diuretic, formula for, 492.
Dogwood, 754.
Dover's powders, improved, 21.
Dried beef pulp, 612.
Dropsy, pill for, 170.
Dry earth dressing, 683.
Dust powder for infants, 683.
Dysentery, 23, 495, 768.
" ipecac enema for, 171, 411.
" ergot in, 495, 753.
Dysmenorrhœa, 350, 601.
" pills for, 412.
Dyspepsia, 29, 107, 700, 122, 755.
" acid, 107.
" bulermic, 157.
" flatulent, 235.
" arsenic in, 359, 424.
" a new remedy for, 489.

Ear, vegetable growth in, 756.
Eclampsia—convulsions, variety epileptic, by A. S. Payne, M. D., 590.
" 608.
Eczema, oil of cade in, 309.
" chloral in, 621.
" infantile, formula for, 37.
Editorials, 32, 54, 59, 116, 181, 245, 248. 369, 380, 432, 498, 564.
Effects of railroad travel upon the health of women, by Ely Van de Warker, M. D., 193.
" of bromide of potassa, 234.
" of opium on snake-bites, 480.
" of diet and exercise, 762.
Electro magnatism in chronic diseases, by A. Donald, M. D., 652.
Elixir of quinine ferri et strichnia phosphatis, 693.
Emmenagogue, uva ursa in, 175.
" formula for, 410, 664.
Emetia, 611.
Enlarged prostrate, by Geo. F. Taylor, M. D., 337.
" " suppressed urine, by J. C. Kurt, M. D., 446.
" gland, 607.
Enlargement of spleen, hypophosphites in, 620.
Enurosis, treatment of, 605.
Epithelioma remov'd by bromine 38, 688
Epilepsy, management of, 151.
" bromide of potassa in, by J. S. Wharton, M. D., 301.
" nitrate of amyl in, 425.
" calabar bean in, 612.
Epileptiform ticdoloreux, 618.
Epistaxis, method of arresting, 648.
Ergotine as a hæmostatic. 47.
Erysipelas, 95.
Ether spray in diagnosis, 176, 686.
Eucalyptus, 606.
" tincture a dressing for wounds, 750
Excision of kidney, 689.
Expectorant mixture, 601.
Explanation of 2 well known symptoms of phthisis, 341.
Exhaustive suppurative discharges, 50.

Feeding bottles, 27.
Fever mixture, aconite, 163.
Fevers, by Dr. T. A. Cooke, 389.
Fissured nipples, 104.
Fistula in ano, 163.
Foot and mouth disease in children, 167
For corneitis, a formula, 25.
For lumbricoides. a formula for, 748.
Foreign correspondence, by A. W. Calhoun, M. D., 11, 656.
Formula in local use, 101,
Fracture in femur of an infant, by A. A. Lyon, M. D., 459.
" base of skull, 686.
Freckles, a remedy for, 168.
From our private prescription book, Dr. J. K. Hodge, 300.
Frozen beef essence, 25.
Functional diseases of the eye, by Swan M. Burnett, M. D., 260, 577.
" medicine, 394.
Furunculus in external meatus, 150.

Gargle, pyrolignous acid, 697.
Gastralgia, formula for, 167, 401.
Gestric irritability, formula for, 606.
Gelseminum in irritable bladder, 109.
General treatment of fevers, by S. S. Satchwell, M. D., 513.
Glandular enlargements, 158.
" swellings, formula for 415, 772
Glaucoma, diagnosis of, 304.

Gleet, 29, 44, 425, 622, 685, 750.
Gleanings from the Minutes of the Medical Society of the city of Wheeling, by S. L. Jepson, M. D., 265.
Glottidis spasmus, chloral in, 754.
Glycerine in putrid sore-throat, 428.
Glycerole of tannin, 613.
Goitre, 616.
" Prof. Gross' treatment, 776.
Grafting with rabbit skin, 694.
Granular lids, 493.
Gray powder, soda and magnesia formula, 47.
Gonorrhœa, treatment of, 309, 683, 496.
" formula for, 480.
" lotion for, 495.
" injection for, 689.
" carbolic acid in, 750.
Gout, chloral in, 368.

Hœmatic, an efficient, 238.
Headache, treatment of, 253.
" sick, bromide of potassium in, 307.
" sick, 701, 157, 695.
Heart-burn, 155, 699.
" murmurs, diagnosis of anæmea 742
" palpitation of, 37.
Hemorrhage, hot water in, 37.
" from teeth, arrest of, 764.
Hemorrhoids, internal, 20.
Hemorrhoids, radical cure of, by A. W. Calhoun, M. D., 278.
" operation on. 343.
" 676.
" Houston's operation, 682.
" ointment for, 693.
Hemaplegia, partial, 164.
Hemoptysis, ergotine hypodermically in, 413.
" 688, 731.
Hemicrania, 768.
Hepatitis, chloride of ammonium in, 368
Hepatic abscess, 368.
" stimulant. 487.
Herpes Zoster, collodion for, 765.
Hiccough, 609.
Hooping cough, 21, 34, 49, 318, 476, 483, 700, 751.
" " aborted, 35.
Hospital gangrene, camphor for, 492.
How to prevent rusting of instruments, 166.
Hydrate chloral, 101.
Hydrochlorate of ammonia, 698.
Hydrathrosis, tincture of iodine in, 481.
Hydrophobia, calomel in, 684.
Hydrocele, cured by seton, 173.
Hydrocele, carbolic acid in, 675.
" 694.
Hypodermie use of strichnia, 151.
" mixture of morphia and strichnia, 477.
" injection, 681.
Hypertrophy of os uteri treated by puncture, 281.
Hyperasthesic of throat, 410.
" stomach, 412.
" testes, 414.
" Heart, 415.
" joints, 419.
" vulva. 691.
Hypophosphites of iron, quinia and strichnia in general debility and nervous exhaustion, 361.
Hysteria, formula for, 417.
Hysterical joint, treatment of, 682.

Ice, 496.
Ileus cured by electricity, 612, 678.
Improved formula, 729.
Imcompatibles with perchlorides of iron, 238.
Incontinence of urine in children, 600.
" chloral in, 692.
Indigestion and its management, 668.
Infantile wasting, 22.
" remittent fever, 30.
Infant's diet, 600.
" the slaughter of, 773.
Ingrowing toe-nails, 746.
Injection of air in uterus, 684.
Intestinal obstruction, ice in, 409.
Intemperance, a cure for, 616.
Inunction in diseases of chilaren, 37.
" in newly born infants, 409.
Iodine starch poultice, 29, 491.
Iodoform, 482.
Irritability of stomach, 35.
Iron in uterine injection, 150.

Kidneys, inflammation cured by application of tar, 760.
Keloid, iodide potassa in, 601.

Labor, spinal cupping in tedious, by Dr. G. D. Hodge, 290.
Lavender, water for collyria, 614
Laxative pill, by Dr. R. C. Word, 230, 413, 759.
" a mild pill, 561.
Leeches, to stop bleeding, 685.
Leucorrhœa, foamula for, 402.
Lithrotrity in childron, 679.
Liquid shampoo, 695.

Local application of leaves of stramonium, 406.
Local anæsthetic, by caustic, 611.

Macroty's racemosa, 111.
Magnetic wells, 758.
Mania, hysterical treatment of, 297.
Measles, tannin in, 679.
Melancholy, treatment of, 681.
Menorrhagia & leucorrhœa, arsenic in, 150.
" treatment of some forms, 348.
" formula for, 406.
" quinine in, 761.
Mercurial stomititis, 609.
Milk fever, 689.
Mississippi State Med. Association, 282.
Modern treatment of internal inflammation, by W. A. B. Norcom, M. D., 65.
Menobonized camphor, 109.
Morbus coxarius, 686.
Morphia and chloral combined, 680.
Mouth wash, 609.
Mumps, 463.
Myrrh, value of, 752.

Nauseous medicines, 27.
Nausea of pregnancy, 420.
Necrosis with spontaneous resection of lower maxilla, by Ely Van de Warker, M. D., 134.
Neuralgia, 33.
" general, 48.
" constant galvanic current, 403
" 418.
" dental treatment for, 420.
" hysterical, 482.
" of kidney and stomach, 489.
" pills for, 743.
" of testes, 744.
" facial, formula for, 756, 768.
New method, nourishing by rectum, 699.
Nitrate of amyl in colic, 303.
" of silver in local inflammations, 687.
Nitric acid, external use of fuming, 693
Nocturnal emissions, 481.
Notes on mucous disease, 297.
Nux vomica in neurosis, 739.

Obituaries, 766.
Offensive breath, 625.
Oiling urethra in catheterism, 106.
Oil of stillingia, 696.
" stophysognia, 698.
Ointment, red percipitate, 681.
On some preparations of iron, by Geo. D. Hodge, M. D., 17.
On the employment of taxis in strangulated hernia, by C. C. F. Gay, M. D., 83.
" spurious consumption, 288.
" functional and semi-organic diseases of the heart, by John E. Lockridge, M. D., 429.
Opium vs. bromide potassa, 487.
Ophthalmia, 768.
Orchitis, belladonna locally in, 405.
" blisters in, 750.
" 690.
Otorrhœa, sulphocarbolate zinc in, 254.
Otitis, anodyne liniment in, 743.
Ovariotomy, 104.
" case, by Robert Battey, M. D., 385.
Ovarian tumors, electrolysis in, 606.
Oxaluria, 21.
Oxalates in urine, formula for, 24.
Oxalate of potassa in peritonitis, 629.
Ozena, treatment of, 363.
Ozokerit, 741.

Perchloride of iron and manganese in necrosis fistulous sinues and hydrocele, 41.
" iron in post-partum hemorrhage, 404.
Penetrating wounds of knee-joint, 623.
Peritonitis, 108, 612, 700.
" following use of speculum, 763
Pessary in offensive uterine disorders, 402.
Phagedenic chancre, 690.
Phenol sodique, 610.
Phlegmasia dolens, sulphate iron for, 172, 691.
Phlegmon of umbilicus, 622.
Phosphorized ether in depression, 313.
Phosphorus, antidote for, 490.
Phosphate of soda, 758.
Phthisis, is it contagious, 421.
" etiology of, 561,
Physical signs of the chest, by W. L. Lipscomb, M. D,, 206.
Pill of creasote, 102.
" carbolic acid, 102.
Pinus canadensis, 488.
Ptyriasis versicola, 24.
Plugging rectum for hemorrhage, 751.
Pluro-pneumonia, 50.
Pneumonia, carbonate ammonia in, 172.
" clinical treatment of, 302.
" quinine in, 411.
" catarrhal, muriate of ammonia in, 484.
" infantile, 488.
" blisters in, 621.

Pneumonia, veratria in, 625.
Podophyllin, 110.
Poisoning by ivy' 462.
" " sorrel, 684.
Popliteal aneurism cured by flexion, 170
Post-partum detetic treatment, 315.
Powder stomachique, 282.
Preservation of tr. kino, 405.
Prescription for jaundice, 48.
Psoas cured by iodide potassa, 687.
Prurin, 696.
Puerperal convulsions, 19, 39, 41, 682.
" " by Dr. Geo. F. Taylor, 233.
Putrid soro throat, glycerine in, 312.
Pyrosis, 764.

Quinine and veratrum incompatible, 27.
" in spasms with hooping cough, by Dr. Thomas Mayo, 336.

Rachitis, observations on, 166.
Railway air cushions, 755.
Reagent for blood, 696.
Recent treatment in aural diseases, 292.
Remedy for dandruff, 100.
Remarks on neuralgia, by Rob't Robson, M. D., 138.
" on old remedies, by T. A. Cooke, M. D., 232.
Removal of plaster paris bandages, 623.
Reports of some interesting cases from the Surgical Clinics of Prof. Largenback, of Berlin, Prussia, by A. W. Calhoun, M. D., 143.
Resorption of fatty tumors, by John E. Lockridge, M. D., 713.
Rheumatic ophthalmia, 35.
Rheumatism, a speedy cure for, 491.
" 608, 418.
" chronic, kerosene in, 665.
" and cider, 750.
" acute, 49, 362.
Rhubarb bitters, 701.
Rigid os, chloral for, 429.

Salivation, 679.
Santonine in eye diseases, 428.
Scarlatina, 22, 307, 767.
" formula for 104, 421.
" sulpho cabolate of soda in 106.
" iron in 409.
Scullcap, 769.
Select formula by J. K. Hodge, M. D., 356.
Seminal emissions, formula, 110.
Senaca root, formula for 692.
Sesquichloride of iron as a prophylactic in rheumatism 43.
Seton treatment of tumors, 361.
Skin diseases, formula for 26, 754, 798.
Simple poultice, 605.
Sleeplessness, formula for 404.
" phosphorus in 427.
Sleep-walking, cure for 695.
Sleeping to death, 757.
Small-pox treatment, 31, 159.
Snake-bites, ammonia in 768.
Soda mint, 424.
" action of 695.
Softening in bones of children, formula for 43.
Soothing ointment, 50.
Soothing syrups, 757.
Sore nipples, 757.
Spermatorrhœa, Dr. Foster, 231.
" treatment of 725.
Spinal irritation, 106, 677.
Spleen. treatment of enlarged 248.
Splenetic and acute ague, 365.
Stimulating expectorant, 49.
" hypodermic injections in typhoid fever, 401.
Strangulated hernia, taxis, 417.
Stramonium, smoking, 764.
Styptic, capsicum a, 161.
" cotton, 356.
" curl paper, 615.
" a new, 692.
Sulpho carbolates, 27.
" of soda, 366.
Sulphovinate of soda, 767.
Sulphurous acid lotion in the treatment of contused wounds, 400.
Suppurating glands of the neck treated by drainage, 50.
Suppurative hepatitis. 107.
" sores, mode of using iodine in 238.
Suppuration of half of one lobe cerebellum, 742.
Sympathetic nervous, trouble from tenia 25.
" ulceration, formula for, 157.
Syphilis, 162, 749.
" iodides of ammonia and sodium in, 227.
" iodide salts in, 477.
" secondary, 697.
" and insanity, 751.
Syphilitic ulceration, sulphurous acid in, 424.
" gummatta of lids, 609.
Syrupus cubabæ, 765.

Tape worm, by R. C. Word, M. D., 212.
" formula for, 605, 743.
" koussin in, 614.
" carrots in, 687.
Taraxicum coffee, 154.
Tea drinkers, 766.
Temperature in disease, 681.
Testicle enlarged, 699.
Test for billary acids, 698.
Tetanus, 165, 483, 612, 756.
The use of earth dressing in burns, 40.

The use of acetic acid in conjuntivitis 40
The use of ferrum candens in diseased joints, by A W Calhoun, M D, 466
The relation of asthma, angiua pectoris and gastralgia, 156.
T he teaspoon as a measure, 173.
The secretions as a guide to treatment, 218.
Thermometer in disease, 732.
Thorocentisis, 480.
Tic doloreaux, 779.
Tinct veratrum in pneumonia of children, 30.
Tincture podoyhollum, dandelion and phytoacca, 363.
Tincture iodine bleached, 742.
To expell foreign bodies from laryinx, 32
To administer pills, 363.
To remove tar, pitch and turpentine, 356.
To prevent stains of iodine, 684.
Tonic and alterative, 486.
Tonsils enlarged, chromic actd in, 624.
" " 685.
Toothache, 170, 600. 601, 766.
" in pregnancy, 685.
Trachoma, 494.
Transfusion, 672.
Treatment of psoriasis, 23.
" of tympany, 24.
" capillary bronchitis in children 31.
" hemorrhage from teeth, 34.
" of cholera infantum, 36.
" nerves by galvanism, 125.
" granula opthalmia, by quinine 222.
" chronic rheumatism, 224.
Turpentine in ring-worm, 26.
Turpentine in parasitic disease of head, 39.
" emulsion, 700.
" mixture. 744.
Typhoid fever with bronchitis, 99.
stimulating hyperdermic injections in, 240.
fever, strychnia in, 416.
Typhus fev r, convalesence of 490.
Tympanitis. 496.
Ulcerated sore throat, 32, 236.
Ulcers of leg, 168.
of cornea. 686.
healed by serum, 750.
Unwholesome candies, 32.
Urea in bile, 699.
Urticaria, 415.
Uterine catarrh, formula for 30.
tonic by Dr. J. M. Lewis, 156.
hemorrhage, 170.
affections ergotine, hypodermically in 174.
Uterus, injection of ergot in 171.
Uvula. 767.
Vaccine virus in glycerine 105
Vaginismus 44, 165.
Valuable tonic, formula for 477.
Value of salt, 478.
Vapor of ammonia in hooping cough 112
Varicose veins, 236.
a new operation 316.
Variola, by A. S. Payne. M. D., 257, 321.
sulphite of soda a prophylactic in 419.
to prevent pittting from 478
arbor vitæ in 746.
sulphite of soda in 753.
bath in 755.
Varicocele, an operation for 679.
Venereal sores 682.
Veratrum. viridi in inflammation 313.
Vermifuge, 768,
Vomiting of pregnancy, 160, 605.
formula for 162, 679.
electricity in 368.
iodine for 601.
strichnia for 612.
obstinate 623.
calumba in 769.
Water, therapeutic uses of, 135.
Will sulphur prevent conception, 746.
Worm-mixture, 740.
Xylol, 617.
Zootrophic powder, 610.

### PART III.

### Editorials, Correspondence and Miscellaneous.

☞ Should any of our subscribers or exchanges fail to get THE COMPANION regularly, they will confer a favor by notifying us of the fact. Our Mailing Clerk will faithfully forward THE COMPANION. Subscribers will please notify Post masters that it will reach their offices, if not lost on the way, between the 15th and 20th of every month. Should it fail to come, after a reasonable time, notify the Editors and it will be sent.

☞ We respectfully solicit Original Articles, Reports of Societies, Clinics Home and Foreign Correspondence, News, etc., etc., of interest to medical readers.

☞ To insure publication, articles should be practical, as brief as possible, and carefully written, on one side of the paper. Articles should be received by the 15th of one month to insure publication in the next. ☞ Friends, send in your articles. ☜

☞ All subscribers and others will please write their names, post-offices, county and State *plainly*

### TO OUR SUBSCRIBERS.

We sincerely thank our subscribers for their kind aid, and oftentimes forbearance, in the conduct of THE COMPANION during the year just gone. We have received many words of cheer and hearty approval to stimulate our efforts and to encourage our hearts in journeying along the path of medical journalism. THE COMPANION has won friends on every side.

But sometimes subscribers complain. They ought to do so when wronged and neglected. To the best of our ability we have endeavored to do our subscribers full justice, and to comply strictly with the terms of our contract with them. Nevertheless a few—and only a few—have complained, alone because of the failure of THE COMPANION to pay them its monthly visits. Only one, so far, has requested its distcontinuance for reasons satisfactory to us, but which, we unite with him in hoping, may soon be removed. We shall, therefore, in view of the above facts, regard those of our subscribers, from whom we have not heard, as subscribers still; and will forward them the first number of the second volume. We trust all will feel as Dr. J. D. Tucker, of Jackson, Tennessee, who says, (whose letter appears in another place,): "If it, (THE COMPANION,) maintains its present merit, I shall continue my subscription as long as I practice physic." Not only do we hope to retain our already large list as life-subscribers, but that each one of these may also, carry out the suggestion of Dr. Tucker, by forwarding us a new one, as well.

Our object has been to issue a meritorious medical journal—one fully up to the demands of the wants of our brethren, who are actively engaged in the practice—and to furnish them, monthly, a condensed view of the progress and therapeutical advances in our science. Our large and talented list of Associate and Corresponding Editors cannot be equaled by any journal published, and betokens an unusual degree of merit for the ensuing year, in our original department.

We have endeavored, with "might and main," with "line upon line and precept upon precept," to exhalt the truth, and to give dignity and honor to the profession as measured by the demands and requirements of the code of medical ethics. We have entreated the pure and good men all over the land, to form a common brotherhood, the shibboleth of which shall be adhesion to the principles and morality of the code of ethics. Organzations of good men for the noble purpose of maintaining the honor of medicine—to rescue it from the sacrilegious hands of pretenders and quacks, is the great professional necessity of the times. Let honesty and integrity pervade our ranks and the good men, united, will become a terror to the refractory and rebellious in a profession they seek to dishonor and disgrace. The great heart of the true men is being moved. All over the country we see evidences of vitality as manifested in our medical societies; and men, like Dr. J. M. Taylor, of Corinth, Miss., see the necessity of medical organization to raise our profession "from the whirlpool of quackery."

The very highest type of Christian gentleman, should be the cherished object of the true physician, to attain. Truth, honor, and integrity should mark his footsteps in every walk of life. Such a man, in medicine, can never seek a "policy" to gain unprofessional advantages. The finger of professional scorn would point him out, and the infamy of the act blast him, from one end of the country to the other, under the lashings of a sound medical organization.

Good men will not and cannot cover up the base actions of legalized quacks and disorganizers. What affects the profession in Massachusetts and New York, must damage it in every other State. The whole body must be "sick" or well; and the man who, in the littleness of his microscopic soul, narrows down the value of morality and ethics to his own locality, stripped of the sympathy and honor of a true heart to strike for others, is an ethical-abortion.

The Companion is pledged to defend the truth. It will not clog its steps, by shouldering a "policy," but out of an honest heart, with the best of its ability, will speak the truth and raise aloft the banner of professional honesty and integrity. It will strike in defence of truth wherever it may be assailed, that a glorious profession may be exhalted and its mission of mercy—of "peace on earth and good will to man"—may, hand in hand with religion, bloom and blossom in every land.

In conclusion, we are pleased and gratified to announce to our subscribers and friends that The Georgia Medical Companion is a success—financially and in the hearts of the profession, as evinced by their appreciation of it on every side.

L. B. Bouchelle, M. D.

---

### BOOKS AND PAMPHLETS.

*The Works of Sir James Y. Simpson Bart. Vol. II. Anæsthesia,* Hospitalism, Hermaphroditism, and a proposal to stamp out Small-Pox and other contageous diseases. By Sir James Y. Simpson Bart, M. D., D. C. L., late Professor of Midwifery in the University of Edinbugh. Edited by Sir W. G. Simpson Bart, B. A. Scholar of Gonville and Caius College, Cambridge. New York. D. Appleton & Co., 549 and 551, Broadway, 1872. For sale in Atlanta, by J. J. & S. P. Richards.

This is a posthumous work of the lamented Simpson, edited by his son. The volume before us will be received by the profession of this country as all previous writings of the great Simpson. Dr. Simpson's investigations in connection with anæsthesia are brought to view in this work. To this gifted man, more than to any other individual, is the profession indebted for that great boon to humanity—anæsthesia. His discovery of chloroform, and his advocacy of it in obstetrical as well as surgical practice, marked a new era in midwifery and surgery. The subjects discussed in the volume before us are handled by a master-hand.

In every well-selected medical library the works of Professor Simpson must have a prominent position,—they would be wanting without them.

*Hand-Book of Skin Diseases.*—By Dr. Isidor Neumann, Docent An Der R. R. Universitat in Wien. Translated from the second German Edition, with notes, by Lucius D. Bulkley, A. M.. M. D., Surgeon of the New York Dispensary, Department of Venerial and Skin Diseases, etc., etc. Illustrated with sixty-six wood-cuts. New York, 1872. D. Appleton & Co. For sale by J. J. & S. P. Richards, Atlanta, Ga.

Skin diseases have been, to the general practitioner, almost a sealed-book. Few are willing to give the subject that amount of attention and study neeessary to diagnose even the most common skin diseases. This has been owing, doubtless, to the confusion among dermatologists in their classsifications and frequent charges in this respect. Still the importance of a practical knowledge of the ordinary cutaneous diseases, which constantly occur, should demand the serious and careful study of every practitioner. For this purpose the work under consideration will prove of great value, especially as the German school of dermatology is brought prominenily to view in 'the volume, while it has been made decidedly practical by the notations of the editor.

*Neuralgia and the Diseases that Resemble it.*—By Francis E. Anstie, M. D., (London,) Fellow of the Royal College of Physicians, Honorary Fellow of King's College, London, etc., etc. New York, D. Appleton & Co., 1872. For sale by J. J. & S. P. Richards, Atlanta, Ga.

We have rarely read a more interesting and instructive volume than that of Dr. Anstie, on neuralgia. Indeed, it is the only tretise on the subject that we have ever seen that is of the least practical value. The author has devoted himself to the investigation of various forms of neuralgias and those diseases that resemble them, with a thoroughness which is well-nigh exhaustive. The chapter on treatment is, of itself, worth more than the price of the work. We advise our friends everywhere to secure a copy of this invaluable work.

*Transactions of the Second Annual Session* of the Medical Society of Virginia, 1871.

We are indebted to the Recording Secretary, Dr. Langdon Edwards, of Lynchburg, for the above Transactions. Our medical brethren of Virginia have placed the Medical Society of Virginia upon the firmest and soundest professional basis. We hail with real satisfaction and pleasure, this exhalted appreciation of the honor and integrity of the profession by our noble brethren of Virginia. The address of the retiring President, Robt. S. Payne, M. D., of Lynchburg, Va., (one of the Associate Editors of THE COMPANION) is worthy of the head and heart of that accomplished and polished physician. The sentiments are pure and his zeal in promoting the organization of the medical gentlemen of his State, should meet the favor of his professional brethren throughout the Old Dominion.

Valuable contributions were read: Dr. John Hurbert Claiborn on dysmenorrhœa; Dr. Alfred G. Tebault, on paludal fever; Dr. A. M. Fauntleroy (President elect), on typhoid fever; Dr. Frederick Horner, Jr., on the cattle disease; Dr. W. J. Gray, case of rheumatism; Dr. W. W. Parker, hypodermic method of treatment. Also synopsis of the discussion by the members of the Society, on inflammation.

We shall, at some future time cull some of the valuable thoughts from the for these use of our readers.

The Society had under consideration the proposition to establish a Medical Journal as the organ of the same. We would hail with pleasure the time when each State could thus own and support a Medical Journal. It would be the highest evidence of the unity and liberality of the profession in each State. But until a new zeal can be invoked and medical men brought in harmonious action, we fear nothing can be accomplished in this direction. Would it not then be better to ingnore State lines for the present, and harmonise upon one journal promotive of the interests of all alike?

